# How well do RNA-Seq differential gene expression tools perform in a complex eukaryote? A case study in *Arabidopsis thaliana*

**DOI:** 10.1093/bioinformatics/btz089

**Published:** 2019-02-06

**Authors:** Kimon Froussios, Nick J Schurch, Katarzyna Mackinnon, Marek Gierliński, Céline Duc, Gordon G Simpson, Geoffrey J Barton

**Affiliations:** 1 Division of Computational Biology, University of Dundee, Dundee, UK; 2 Centre for Gene Regulation and Expression, School of Life Sciences, University of Dundee, Dundee, UK; 3 GReD, Faculté de Médecine 28, place Henri Dunant BP 38 - 63001 CLERMONT-FERRAND; 4 Division of Plant Sciences, School of Life Sciences, University of Dundee, Dundee, UK

## Abstract

**Motivation:**

RNA-seq experiments are usually carried out in three or fewer replicates. In order to work well with so few samples, differential gene expression (DGE) tools typically assume the form of the underlying gene expression distribution. In this paper, the statistical properties of gene expression from RNA-seq are investigated in the complex eukaryote, *Arabidopsis thaliana*, extending and generalizing the results of previous work in the simple eukaryote *Saccharomyces cerevisiae.*

**Results:**

We show that, consistent with the results in *S.cerevisiae*, more gene expression measurements in *A.thaliana* are consistent with being drawn from an underlying negative binomial distribution than either a log-normal distribution or a normal distribution, and that the size and complexity of the *A.thaliana* transcriptome does not influence the false positive rate performance of nine widely used DGE tools tested here. We therefore recommend the use of DGE tools that are based on the negative binomial distribution.

**Availability and implementation:**

The raw data for the 17 WT *Arabidopsis thaliana* datasets is available from the European Nucleotide Archive (E-MTAB-5446). The processed and aligned data can be visualized in context using IGB (Freese *et al.*, 2016), or downloaded directly, using our publicly available IGB quickload server at https://compbio.lifesci.dundee.ac.uk/arabidopsisQuickload/public_quickload/ under ‘RNAseq>Froussios2019’. All scripts and commands are available from github at https://github.com/bartongroup/KF_arabidopsis-GRNA.

**Supplementary information:**

Supplementary data are available at *Bioinformatics* online.

## 1 Introduction

Short read RNA sequencing (RNA-seq) has become the method of choice for transcriptome-wide quantification of gene expression and the analysis of differential gene expression (DGE) between experimental conditions (Mortazavi *et al.*, 2008; Nagalakshmi *et al.*, 2010). RNA-seq data analysis typically involves aligning short sequence fragments (reads) to a reference genome or transcriptome or assembling them *de novo*, counting the resulting alignments that fall within an annotated feature region or a contig, then identifying any significant differences between two or more conditions. More than a dozen computational tools have been developed to identify Differential Expression (DE) from RNA-seq data and each makes assumptions about the nature and behavior of the expression data (Anders and Huber, 2010; Frazee *et al.*, 2014; Hardcastle and Kelly, 2010; Law *et al.*, 2014; Leng *et al.*, 2013; Li and Tibshirani, 2013; Li *et al.*, 2012; Love *et al.*, 2014; Lund *et al.*, 2012; Moulos and Hatzis, 2015; Ritchie *et al.*, 2015; Robinson *et al.*, 2010; Tarazona *et al.*, 2011; Trapnell *et al.*, 2012; Wang *et al.*, 2010). Based on these assumptions, the tools calculate the probability that two sets of measurements come from the same statistical distribution, thus determining whether a genuine shift in expression is a more likely explanation for the observed values than random chance. Incorrect assumptions can lead to poor false discovery rate (FDR) control and inaccurate true positive identification in the DE calls. Such errors will propagate downstream into the biological interpretation of the DE results. Although DGE methods are increasingly being used to identify DE of other genomic regions (i.e. exons, spliced transcripts, etc.) (Frazee *et al.*, 2014; Gaidatzis *et al.*, 2015; Wood *et al.*, 2013) the tools are most commonly used to identify DE for genes (DGE) which is the focus of this paper.

Several studies have assessed the performance of DGE tools (Bullard *et al.*, 2010; Busby *et al.*, 2013; Frazee *et al.*, 2014; Law *et al.*, 2014; Leng *et al.*, 2013; Li and Tibshirani, 2013; Li *et al.*, 2012; Love *et al.*, 2014; Lund *et al.*, 2012; Moulos and Hatzis, 2015; Rapaport *et al.*, 2013; Ritchie *et al.*, 2015; Seyednasrollah *et al.*, 2015; Soneson, 2014; Trapnell *et al.*, 2012). However, these studies were carried out using either simulated data or biological data that was originally designed for a different purpose. Although a few of these studies have explored high biological replication by leveraging publicly available datasets on individuals within a species (Bottomly *et al.*, 2011; Burden *et al.*, 2014; Guo *et al.*, 2013; Seyednasrollah *et al.*, 2015; Soneson and Delorenzi, 2013), most have a limited level of replication. Recently, a study was performed in yeast (*Saccharomyces cerevisiae*) specifically designed to test both the underlying statistical properties of RNA-seq data across biological and technical replicates and the influence of replication on DGE results (Gierlinski *et al.*, 2015; Schurch *et al.*, 2016). With 48 biological replicates per condition, it investigated the distribution of read counts per gene across biological replicates and the relationship between the replication level, the FDR and the discoverable effect size for 11 different DGE tools. However, most *S.cerevisiae* genes do not contain introns so it is unclear whether the conclusions of Schurch *et al.* (2016) hold true for complex transcriptomes where splicing is widespread and leads to alternative isoforms from the same gene locus.

In this paper, RNA-seq data from 17 wild-type (WT) biological replicates of *Arabidopsis thaliana* were used to explore read count measurements across replicates and the FDR of DGE tools. Although *A.thaliana* has a relatively small genome, its transcriptome is similar in scale and complexity to that of human and model mammal species (Arabidopsis Genome Initiative, 2000; Carvalho *et al.*, 2013; Krishnakumar *et al.*, 2015) and its genome is extensively annotated. Accordingly, conclusions from the high-replicate RNA-seq study presented here should provide useful guidance for work in other complex eukaryotes as well.

## 2 Materials and methods

### 2.1 Sample preparation and sequencing

The RNA-seq data for this study are WT *A.thaliana* Colombia-0 (Col-0) biological replicates from three separate experiments (hereafter ExpA, ExpB and ExpC). Briefly, for all three experiments WT *A.thaliana* Col-0 seeds were sown aseptically on MS10 plates. The seeds were stratified for 2 days at 4°C and then grown at a constant 21°C under a 16-h light/8-h dark cycle for a further 14 days, at the end of which the seedlings were harvested. Total RNA was isolated from the seedlings with the RNeasy Plant Mini Kit (Qiagen) and treated with TURBO^TM^ DNase (Ambion). An aliquot of 4 μl of ERCC spike-ins (External RNA Controls Consortium, 2005) at a 1:100 dilution were added to 1 μg/6 μl of total RNA. Libraries were prepared using the Illumina TruSeq Stranded Total RNA with Ribo-Zero Plant kit. The libraries were sequenced on a HiSeq2000 at the Genomic Sequencing Unit of the University of Dundee. Two of the experiments, ExpA and ExpB, have seven biological WT replicates (replicates 1–7 and 8–14, respectively) while ExpC has 3 (replicates 15–17), for a total of 17 biological WT replicates and ∼$1.7\times 10^{9}$ 100-bp paired-end reads across the three experiments. The plants were sown, grown, harvested and the libraries were prepared by the same lab, and the sequencing was performed on the same machine by the same people at the same sequencing facility and all the samples included the ERCC spike-ins which can verify that the WT samples are consistent and comparable across experiments.

### 2.2 Quality control, alignment and quantification

The quality of the data was quantified using *FastQC* (Anders, 2010, available at http://www.bioinformatics.babraham.ac.uk/projects/) v0.11.2 with all the replicates performing as expected for high quality RNA-seq data with excellent median per-base quality (≥38) across >90% of the read length. The read data for each sample were aligned to the TAIR10 *A.thaliana* genome assembly using the splice-aware aligner *STAR* v2.5.0a (Dobin *et al.*, 2013). The index was built with parameter ‘*–sjdbOverhang 99’* and the alignment was run with parameters: ‘*–outSAMstrandField intronMotif –outSJfilterIntronMaxVsReadN 5000 10000 15000 20000 –outFilterType BySJout –outFilterMultimapNmax 2 –outFilterMismatchNmax 5’*.

Read counts per gene were then quantified from these alignments with *featureCounts* [v1.4.6-p4 (Liao *et al.*, 2014)], excluding reads with ambiguous assignments, multi-mapping reads and multi-overlapping reads, using the publicly available Araport11 annotation (pre-release December 3, 2015, comprising 33, 851 genes) (Krishnakumar *et al.*, 2015) with the parameters: ‘*-t exon -g gene_id -s 2 -p –P’*.

These read counts were used without further processing to examine the false positive (FP) performance of nine DGE tools, allowing each tool to carry out its default normalization. The tools were used in the R v3.2.2 environment (R Development Core Team, 2011) and installed through Bioconductor v3.2.

For the purposes of comparing the expression distribution models, consistently normalized data was required. As some of the distributions in question are discrete, normalized integer read counts were used for this purpose, which were calculated by randomly down-sampling read-pairs from each replicate to the level of the replicate with the lowest read depth. In this study, the focus is on the collective behavior of gene expression, rather than the biological interpretation of the expression of any specific gene, so this type of normalization is appropriate here. However, it is not recommended for typical gene expression analysis studies, as some low-expression signals can randomly be lost during resampling.

After the normalization, each replicate consisted of $∼77\times 10^{6}$ read-pairs, which were then aligned to the genome and quantified using the same steps described above.

### 2.3 Performing the tests

The read counts of each gene were tested against four theoretical distributions across replicates: normal, log-normal, Poisson and negative binomial. For the normal and log-normal distributions the goodness-of-fit was determined using the test for normality from D’Agostino *et al.* (1990). This approach to testing the log-normal distribution cannot be applied to data containing zeroes, ruling out this test for ∼23% of genes. The expression data for all genes, including those with zeroes, was tested for consistency with a Poisson distribution using a *χ^2^* test (Fisher, 1950) and for the negative binomial distribution, the method described by Meintanis (2005) was employed. Briefly, the method described by Meintanis (2005) is based on the probability generating function and because the distribution of the test statistic is not known in closed form it requires a bootstrap to calculate *P*-values. This makes it computationally expensive and limits its sensitivity. In this case we perform 10^7^ bootstraps resulting in *P*-values that are limited to be ≳10^−7^. In each case, rejection of the null hypothesis was based on a Benjamini–Hochberg corrected critical *P*-value of 0.05 (Benjamini and Hochberg, 1995).

In order to test the FPs control of the DGE tools, two sets of $n_{r}$ replicates were randomly selected without replacement from the pool of 16 ‘clean’ WT replicates (see Section 3.1). By using two sets of real biological replicates from the same condition, we are measuring the performance of the tools when the null hypothesis is explicitly true, free of the confounding complications that would occur from using simulated data or two biological conditions for which the ground truth cannot be known with certainty. DGE was then called on each of the set pairs with each of nine DGE tools (Table 1). Since the choice of normalization does not affect the outcome dramatically (Dillies *et al.*, 2013; Trapnell *et al.*, 2012) and forcing a uniform method across all tools is not supported by all tools and may lead to inappropriate processing of the data, each tool was allowed to apply its default normalization. Since all sets are drawn from the same WT pool, every gene identified as significantly differentially expressed is, by definition, a FP. This process was repeated 100 times for each sample size in the range ${3\leq n}_{r}\leq 7$ for each tool.

**Table 1.** RNA-seq DGE tools used in this study

Name	Assumed distribution	Normalization	Description	Version	Citations^a^
*baySeq* (Hardcastle and Kelly, 2010)	Negative binomial	Internal	Empirical Bayesian estimate of posterior likelihood	2.4	259
*DEGseq* (Wang *et al.*, 2010)	Binomial	None	Random sampling model using Fisher's exact test and the likelihood ratio test	1.24.0	748
*DESeq* (Anders and Huber, 2010)	Negative binomial	DEseq	Shrinkage variance	1.22.0	4308
*DESeq2* (Love *et al.*, 2014)	Negative binomial	DEseq	Shrinkage variance	1.10.0	4277
*EBSeq* (Leng *et al.*, 2013)	Negative binomial	DEseq (median)	Empirical Bayesian estimate of posterior likelihood	1.10.0	301
*edgeR* (Robinson *et al.*, 2010)	Negative binomial	TMM	Empirical Bayes estimation and either an exact test analogous to Fisher’s exact test but adapted to over-dispersed data or a generalized linear model	3.12	5339
*Limma* (Ritchie *et al.*, 2015)	Log-normal	TMM	Generalized linear model	3.26.2	2197
*Poisson-Seq* (Li *et al.*, 2012)	Poisson log-linear model	Internal	Score statistic	1.1.2	80
*SAM-Seq* (Li and Tibshirani, 2013)	None	Internal	Mann–Whitney test with Poisson resampling	2.0	136

*Note*: A list of the DGE tools and their respective versions used in this study, together with their core methodology. The number of citations is shown as proxy for each tool’s popularity.

a Citations as reported by PubMed Central: number of articles that reference the listed source on January 28, 2019.

## 3 Results

### 3.1 Consistency among replicates

**Fig. 1.**
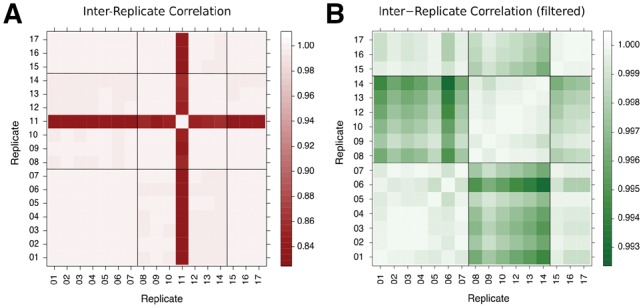
Pairwise inter-replicate Pearson’s correlation of gene expression. The black grid lines indicate the grouping of the replicates with regards to the three experiments. (**A**) Correlation matrix of gene expression for all 17 replicates. Apart from replicate 11, all replicates correlate very well. (**B**) Same as left, but with replicate 11 filtered out, allowing the patterns of correlation among the remaining 16 replicates to be better seen

Our dataset consisted of 100-base reads from 17 WT *A.thaliana* samples with sequencing throughput of at least $77\times 10^{6}$ read-pairs per sample. The samples were collated from three separate experiments, but otherwise had been processed in identical ways in terms of personnel and equipment to minimize confounding factors. The global gene expression measurements from 16 of the 17 WT biological replicates are well correlated, irrespective of the different experiments (*R* > 0.99, Fig. 1). Replicate 11 correlates less well with all the other replicates (0.83≤R ≤ 0.87, Fig. 1A) and so was excluded from subsequent analysis. Removal of ribosomal RNA was incomplete in some samples, evidenced by high read counts for ribosomal genes (Supplementary Fig. S1 and Table S2) and a high level of multi-mapping reads (Supplementary Table S2). However, excluding reads mapping to ribosomal RNA genes in the remaining replicates does not strengthen the inter-replicate correlations (Supplementary Fig. S3). A low level of uniform read coverage across the genome was observed in replicates 8–14, all belonging to the same experiment, explaining the marginally lower correlation between the replicates of this experiment and the other replicates (Fig. 1B).

**Table 2.** Fraction of genes whose cross-replicate expression distribution rejects the null hypothesis for each of four distribution models

Replicates	Poisson (%)	Normal (%)	Log- normal (%)	Neg. Binomial (%)
(i)	70	23	2	0
(ii)	65	10	0	0
(iii)	59	9	0	0

*Note*: Cases: (i) all replicates 1–17, excluding the contaminated replicate 11 (see also Fig. 2), (ii) only the non-noisy replicates 1–7 and 15–17 and (iii) replicates 8–10 and 12–17 as control for statistical power.

### 3.2 Distribution of gene read counts across replicates

**Fig. 2.**
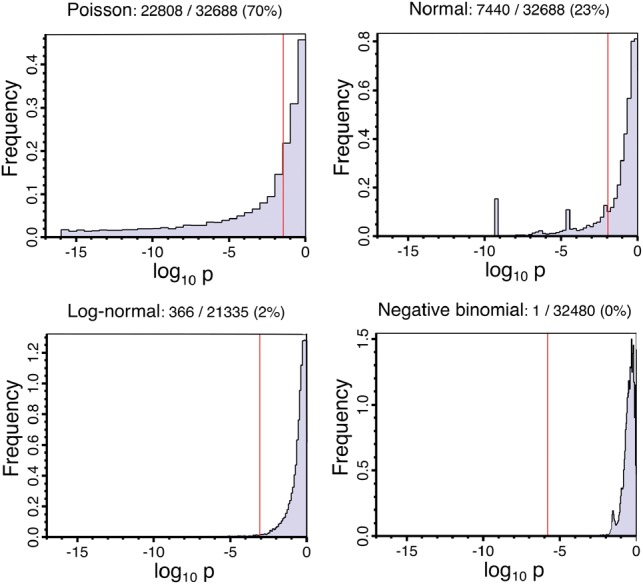
Inter-replicate variation goodness-of-fit. Histograms of the probability that the genes’ fragment counts across replicates are compatible with each of the four specified distributions. The fraction of genes rejecting the distribution model is given above each plot. The Benjamini–Hochberg adjusted critical *P*-value is shown in red

Figure 2 shows the results of the goodness-of-fit test against three model distributions, performed for each gene, across all replicates. The negative binomial null hypothesis is rejected at the pP = 0.05 level by only one gene, while the log-normal null hypothesis is rejected for 2% of genes. In contrast, the normal and Poisson null hypotheses are rejected for 23 and 70% of genes, respectively.

**Fig. 3.**
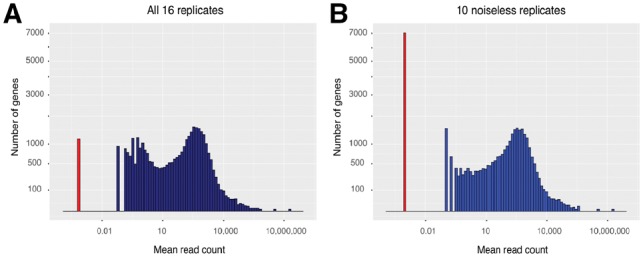
Distribution histogram of gene expression. Each gene is represented by the mean of its read count estimates across replicates. The various levels of non-zero expression are shown in blue. The *x*-axis here is logarithmic, so genes with zero expression were added manually at an arbitrary but distinct location on the axis (red bar). The *y*-axis is square-root scaled

As mentioned above, replicates 8–14 presented a low level of uniform read coverage along the genome. We believe this to be noise consistent with a small amount of genomic DNA contamination in the affected samples. Such reads have the potential to interfere with the fitting of statistical distributions to the data, as they can make silent genes artificially appear as expressed. Indeed, Figure 3 shows that ∼6000 of the genes annotated in Araport11 appear to be lowly expressed in the affected replicates, but are not detected in the ten replicates from the other two experiments (replicates 1–7 and 15–17). The potential for this noise to impact on the distribution measurements was assessed by comparing the fraction of genes that reject the null hypothesis for each of the distributions using (i) the filtered dataset (replicates 1–10 and 12–17), (ii) replicates 1–7 and 15–17 only and, as a control and (iii) replicates 8–10 and 12–17. The rejection rates for each of the null hypotheses are summarized in Table 2. For tests against the negative binomial or log-normal distributions, the fraction of genes that rejects the null hypothesis in each set is similar irrespective of the replicate selection. For tests against the normal distribution, reducing the number of replicates used (cases ii and iii) reduces the fraction of the genes that reject the null hypothesis from 23 to ∼10% irrespective of whether the excluded replicates were the noisy set or the control. The lack of difference between excluding the noisy or control replicates demonstrates that the apparent improvement of model fit is due to the reduced statistical power of the tests because of the smaller number of replicates rather than to an improvement in signal to noise ratio from excluding the noisy replicates. We conclude that the apparent low-expression noise in replicates 8–14 is not unduly influencing our conclusion with regards to the goodness-of-fit distribution tests. Similarly, excluding reads that map to rRNA genes in the replicates does not affect the results of the goodness-of-fit distribution tests.

### 3.3 FP behavior of DGE tools

In this section we test the FP performance of DGE tools by performing DGE tests with samples drawn from the same biological condition. Since, in this case, the null hypothesis is explicitly true, this test should return no differentially expressed genes and, thus, every gene flagged as differentially expressed is a FP. We note that this is intentionally not a test of equivalence and is not intended to test whether the two sets of samples are the same. Instead, it mirrors a real-world scenario in which a researcher is testing for DGE between two biologically meaningful conditions where, unknown to the researcher, there is no true difference in gene expression. One such example would be a comparison between treated samples and control samples in an experiment where the treatment was not effective.

**Fig. 4.**
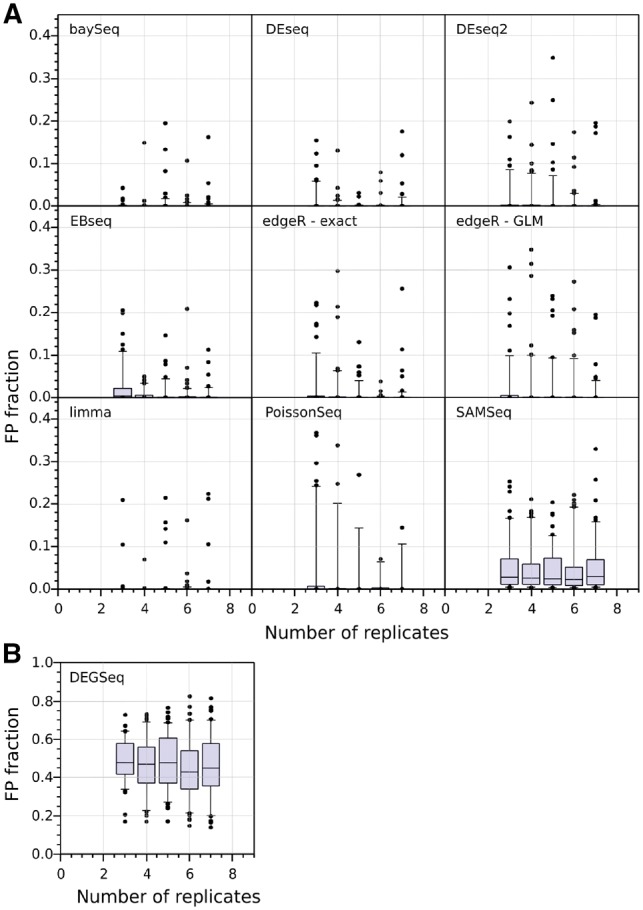
FP fractions in WT versus WT comparisons of DGE. A total of 100 bootstrap iterations performed for each value out of a range of sample sizes from 3 to 7 replicates per condition. The plots show the median (horizontal line), quartiles (shaded blue boxes), 95% data limits (capped vertical lines) and outliers (black points) for the fraction of bootstraps in which a gene was called as differentially expressed (without any fold-change threshold). Panel (**A**) and (**B**) differ on the range of the *Y*-axis. *DEGSeq* displays poor FP performance (nearly 50% of its positives are false). The performance of the tools is a result of their choice in methods and models (Table 1), with the lowest FP tools using the negative binomial or log-normal distributions

The distribution of the FP fraction as a function of the number of replicates, bootstrapped 100 times for each DE tool (SGE genes identified with FDR < 0.05, no minimum fold-change threshold), is shown in Figure 4. Most tools consistently control their FP fraction well at all numbers of replicates despite the presence of a small number of outlier results. *DEGseq* fails to control its FP fraction adequately, likely due to over-estimation of the number of significantly differentially expressed genes. Finally, although the median FP fraction for *SAM-seq* is <5%, its performance is worse than the other tools at all the tested numbers of replicates, suggesting that it is a poorer choice for calling DGE.

## 4 Discussion

In this study, the statistical assumptions made by tools that identify DGE from RNA-seq read count data were validated in a high-replicate experiment, in the context of *A.thaliana*, a higher eukaryote with a complex transcriptome. This work extends our previous observations about the properties of RNA-Seq data in 48 replicates from WT and mutant (Δ*snf2*) *S.cerevisiae* (Gierlinski *et al.*, 2015; Schurch *et al.*, 2016) and provides evidence that the same properties are present in *A.thaliana* and so are likely to be generally valid for RNA-seq in eukaryotes. The 17 true biological replicates studied here are consistent with the recommendations of our previous 48 replicate study which suggested that 6–12 replicates should be sufficient for most RNA-seq studies (Schurch *et al.*, 2016). With 17 replicates, this study is currently the most highly replicated high-coverage full-transcriptome RNA-Seq dataset for a higher eukaryote. The clear difference in the results of the goodness-of-fit-tests between the normal and Poisson distributions and the negative binomial and log-normal distributions demonstrates that this dataset has sufficient power to distinguish between these different distributional models. The dataset should prove a useful resource for *A.thaliana* biology as well as a benchmarking dataset for tool developers.

In this study we focused on a single strain of *A.thaliana*. Our findings show that the negative binomial and log-normal distribution are both good choices as models for the cross-replicate variability of RNA-seq read counts. We also studied the FP performance of DGE tools using two sets of replicates drawn from the same condition. An alternative approach would be to use artificial datasets (simulations), but such datasets can confound the analysis by introducing the assumptions and biases built into the simulation. The study demonstrates that six out of the nine DGE tools examined here control their identification of FPs well even with only three replicates. These tools (*baySeq, DEseq, DEseq2, EBseq, edgeR, limma*) are based on the negative binomial or log-normal distributions and employ a variety of normalization strategies. In contrast, the non-parametric *SAM-seq*, the Poisson-based Poisson-seq and, in particular, the binomial-based *DEGseq* do not control FPs well.

Our results reinforce the conclusions previously reached by our study of the yeast transcriptome. The transcriptome of *A.thaliana* is considerably more complex than *S.cerevisiae*, with almost four times the number of protein-coding genes (27 667 in *A.thaliana*, 7126 in *S.cerevisiae*) and widespread alternative splicing and alternative polyadenylation. The similarity of the results from these two very diverse organisms lends themselves to the hypothesis that the conclusions of both studies regarding the expression distributions and the tool performance are extendable to a wide range of eukaryotes.

The concept of gene expression in complex transcriptomes is confounded by the presence of alternative transcript isoforms, which give the organism additional means to regulate a gene’s expression. This type of regulation is not necessarily reflected in changes to the total transcriptional output of a gene. Ideally, expression studies should aim to quantify the abundance of alternative isoforms individually and independently. Interestingly, the sum of independent random variables with a negative binomial distribution itself has a negative binomial distribution. Thus, our finding that a negative binomial is a suitable model for gene expression variability across replicates is consistent with the hypothesis that the underlying variability of expression of the individual isoforms also follows the negative binomial distribution. If this is true, tools originally intended for the study of DGE may also be appropriate for studying differential transcript expression.

In summary, our analyses show that the statistical properties of gene expression are similar between a simple and a complex model eukaryotic organism, and validate the model assumptions of the best-performing DGE tools.

## Supplementary Material

btz089_Supplementary_Data
