# Supplementary material for: How well do RNA-Seq differential gene expression tools perform in a complex eukaryote? A case study in Arabidopsis thaliana
Source: Bioinformatics. 2019 Feb 6;35(18):3372–7. doi: 10.1093/bioinformatics/btz089 (PMC6748783; doi:10.1093/bioinformatics/btz089)
Supplement: btz089_Supplementary_Data [file btz089_supplementary_data.zip › btz089-suppl_data/S3-Fig.pdf]

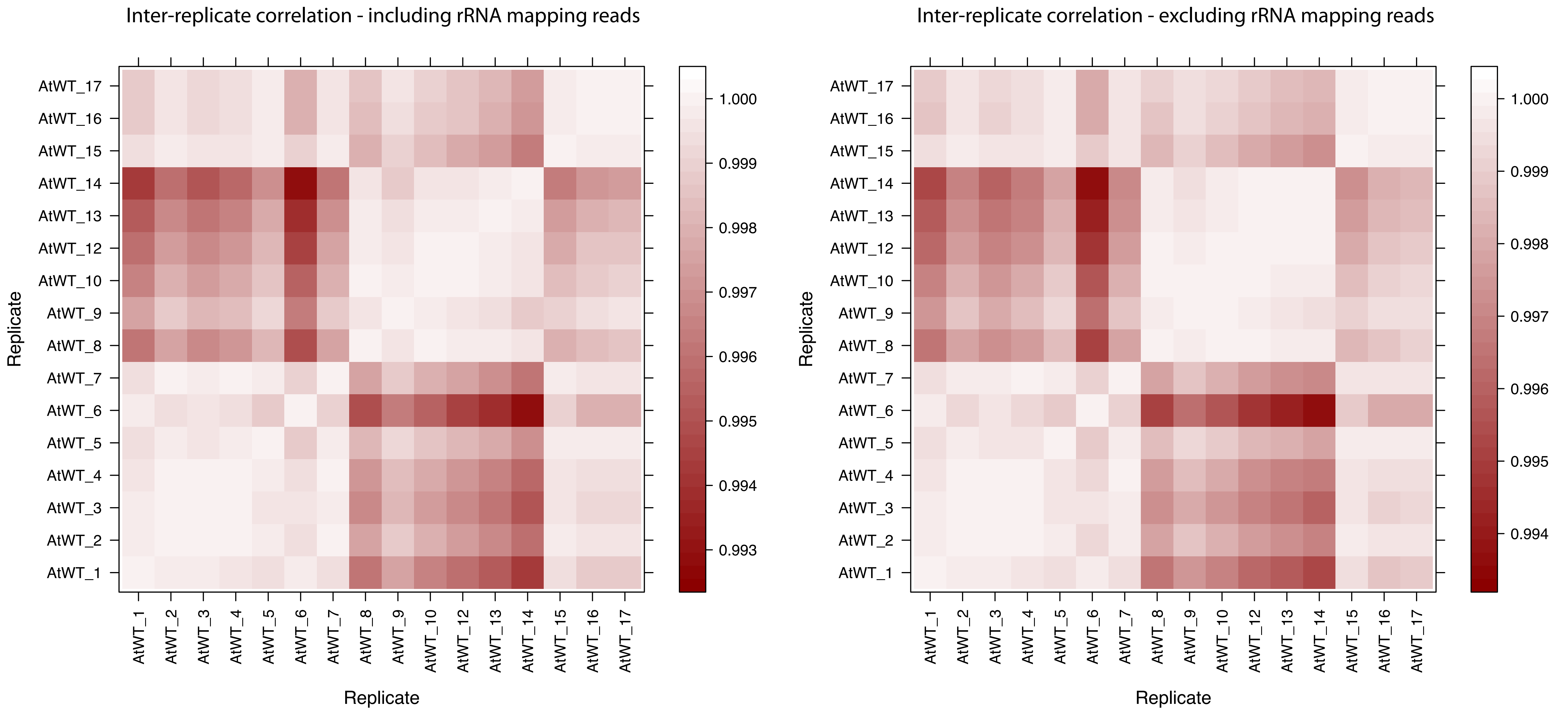
Inter-replicate correlation - including rRNA mapping reads
Inter-replicate correlation - excluding rRNA mapping reads
AtWT_17
AtWT_16
AtWT_15
AtWT_14
AtWT_13
AtWT_12
AtWT_10
AtWT_9
AtWT_8
AtWT_7
AtWT_6
AtWT_5
AtWT_4
AtWT_3
AtWT_2
AtWT_1
1.000
0.999
0.998
0.997
0.996
0.995
0.994
0.993
Replicate
