# Supplementary material for: How well do RNA-Seq differential gene expression tools perform in a complex eukaryote? A case study in Arabidopsis thaliana
Source: Bioinformatics. 2019 Feb 6;35(18):3372–7. doi: 10.1093/bioinformatics/btz089 (PMC6748783; doi:10.1093/bioinformatics/btz089)
Supplement: btz089_Supplementary_Data [file btz089_supplementary_data.zip › btz089-suppl_data/Supplimentary Information Captions.docx]

**S1 Figure. Read counts for the ribosomal genes across the 17 replicates.** Data shown for both the non-normalized and the resampled data sets. Ribosomal genes are strongly expressed in replicate 11, despite having a similar sequencing depth to the other replicates, suggesting that the ribosomal depletion step did not work well for this replicate (see also S2 Table). Araport rRNA gene classification: 4.5S - ATCG00960, ATCG01170. 5S - ATCG00970, ATCG01160, ATMG01380. 5.8S - AT2G01020, AT3G41979. 16S - ATCG00920, ATCG01210. 18S - AT2G01010, AT3G41768, ATMG01390. 23S - ATCG00950, ATCG01180. 26S - ATMG00020.

**S2 Table. Alignment summaries for all replicates.** [A] Non-normalised data set. [B] Re-sampled data set. [C] rRNA mapping fractions (non-normalized dataset). [D] Non-normalised rRNA filtered data set. [E] Re-sampled rRNA filtered data set.

**S3 Figure. Comparison of pairwise inter-replicate Pearson’s Correlation of gene expression, including and excluding rRNA mapping reads.** (A): Correlation matrix of gene expression for all 16 replicates (replicate 11 excluded) including rRNA mapping reads. (B): Correlation matrix of gene expression for all 16 replicates (replicate 11 excluded) excluding rRNA mapping reads.

**S4 Figure. Comparison of inter-replicate variation goodness-of-fit results, including and excluding rRNA mapping reads.** Histograms of the probability that the genes’ fragment counts across replicates are compatible with each of the four specified distributions. The fraction of genes rejecting the distribution model is given above each plot. The Benjamini-Hochberg adjusted critical p-value is shown in red. The leftmost panels are the full datasets. The rightmost panels are the same datasets but with the reads from each replicate that map to the 1360 rRNA regions in the Ensembl v40 Arabidopsis thaliana annotation removed prior to down-sampling normalization and analysis.
