# Supplementary figures and images for: How well do RNA-Seq differential gene expression tools perform in a complex eukaryote? A case study in Arabidopsis thaliana
Source: Bioinformatics. 2019 Feb 6;35(18):3372–7. doi: 10.1093/bioinformatics/btz089 (PMC6748783; doi:10.1093/bioinformatics/btz089)

rRNA abundance across replicates

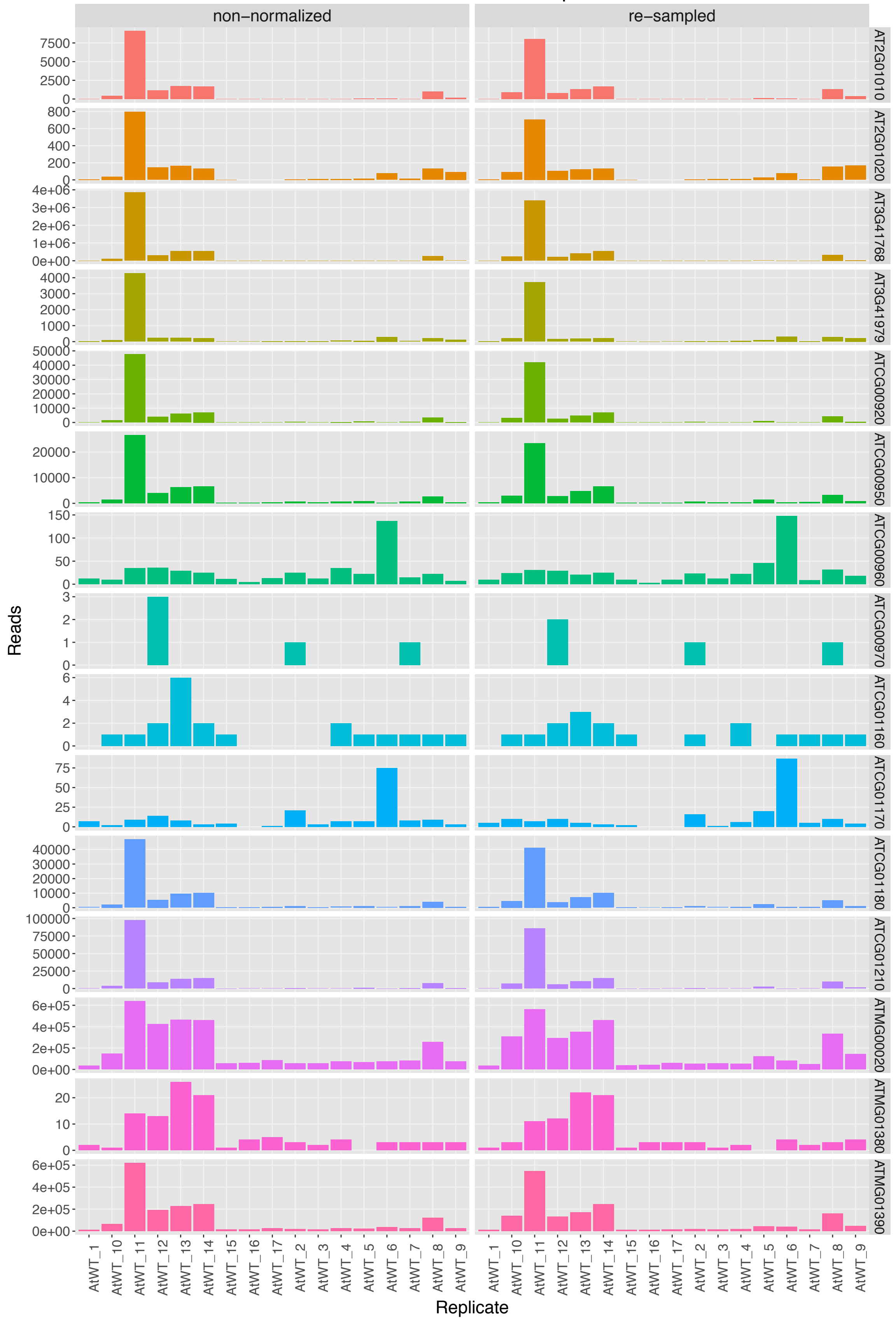

Supplement: btz089_Supplementary_Data [file btz089_supplementary_data.zip › btz089-suppl_data/S1-Fig.pdf]
